# The “Smart + Specialty” integrated model improves emergency efficiency and success in aortic dissection

**DOI:** 10.3389/fcvm.2025.1677574

**Published:** 2026-02-02

**Authors:** Lingling Lai, Zhen Li, Meiping Zhao

**Affiliations:** 1 Nursing Department, Sir Run Run Shaw Hospital, Zhejiang University School of Medicine, Zhejiang, China; 2 Plastic Surgery and Cosmetic Center, Sir Run Run Shaw Hospital, Zhejiang University School of Medicine, Zhejiang, China

**Keywords:** smart healthcare, specialty, integrated care, emergency medicine, aortic dissection, treatment success rate, adverse events

## Abstract

**Objective:**

To explore the impact of the integrated treatment model under the “smart + specialty” concept on the treatment success rate and adverse events in emergency patients with aortic dissection (AoD).

**Methods:**

A total of 166 patients with AoD treated at our hospital from March 2022 to April 2024 were selected and divided into two groups according to the order of treatment. The control group consisted of 81 patients treated from March 2022 to March 2023 and received routine emergency procedures, while the study group included 85 patients treated from April 2023 to April 2024 under the “smart + specialty” integrated treatment model. The treatment success rate, treatment efficiency, incidence of adverse events, and nursing satisfaction were compared between the two groups.

**Results:**

The treatment success rate in the study group was 100% (85/85), which was higher than the control group's rate of 92.59% (75/81), with a statistically significant difference (*P* = .03). The study group had shorter triage time, diagnosis time, emergency stay time, referral time to intensive care unit (ICU), and symptom remission time compared to the control group, with statistically significant differences (*P* < .001). The total incidence of adverse events in the study group was 3.53% (3/85), significantly lower than the control group's rate of 12.00% (9/75), and the total family nursing satisfaction in the study group was 96.47% (82/85), significantly higher than the control group's rate of 87.65% (71/81), with statistically significant differences (*P* = .04).

**Conclusion:**

The “smart + specialty” integrated treatment model can improve treatment efficiency and success rates in emergency AoD patients, reduce the risk of adverse events, and enhance family nursing satisfaction.

## 1 Introduction

Aortic Dissection (AoD) is a life-threatening cardiovascular emergency characterized by rapid onset and progression, with high mortality rates if not promptly treated (1–3). Evidence indicates that the first 90 min after symptom onset represent a critical “golden window” for intervention; without timely treatment, mortality rates rise sharply to approximately 20% within 24 h, 37% by 48 h, and up to 74% within one week (4). Therefore, establishing an efficient and standardized treatment model for AoD is essential to improve patient outcomes.

However, current emergency care pathways for cardiovascular emergencies such as AoD face several systemic challenges. These include delays in patients seeking care, variability in diagnostic and treatment processes, and insufficient integration of pre-hospital and in-hospital information (5–7). These factors can significantly impede treatment timeliness and effectiveness.

In recent years, the integration of information technology into healthcare has led to the development of smart emergency platforms. Such systems enable specialized information management across pre-hospital and in-hospital settings, showing potential to enhance coordination and throughput in time-sensitive emergencies (8–10). Despite these advancements, there remains a scarcity of clinical studies evaluating the implementation and effect of such integrated models in the context of AoD.

Therefore, this study aims to evaluate the impact of a “Smart + Specialty” integrated emergency care model on treatment success rates and adverse events in patients with aortic dissection, with the goal of providing a reference for optimizing clinical pathways.

## 2 Materials and methods

### 2.1 The “smart + specialty” concept

#### 2.1.1 Formation of a specialty emergency team

The team includes attending physicians from the emergency department and vascular surgery, head nurse of the emergency department, emergency nurses, vascular surgery specialty nurses, and pre-hospital emergency nurses. The head nurse of the emergency department serves as the team leader, the vascular surgery attending physician is responsible for consultation and decision-making, the emergency attending physician provides initial treatment and issues surgical orders, emergency nurses prepare equipment, drugs, and instruments, vascular surgery specialty nurses are responsible for specialty nursing care, and pre-hospital emergency nurses handle pre-hospital nursing care. A WeChat group chat is created for team members, with a reminder that only work-related messages should be sent in the group.

#### 2.1.2 Homogenized training and assessment

The team leader is responsible for nursing training, conducting specialized training on the diagnosis, emergency treatment, nursing, and transportation of AoD, clarifying nursing responsibilities, and conducting scenario simulations to enhance nurses’ emergency skills. Theoretical and skill assessments are conducted after training, and only those who pass are allowed to participate in nursing work.

#### 2.1.3 Construction of “smart + specialty” emergency system

**Figure 1:**
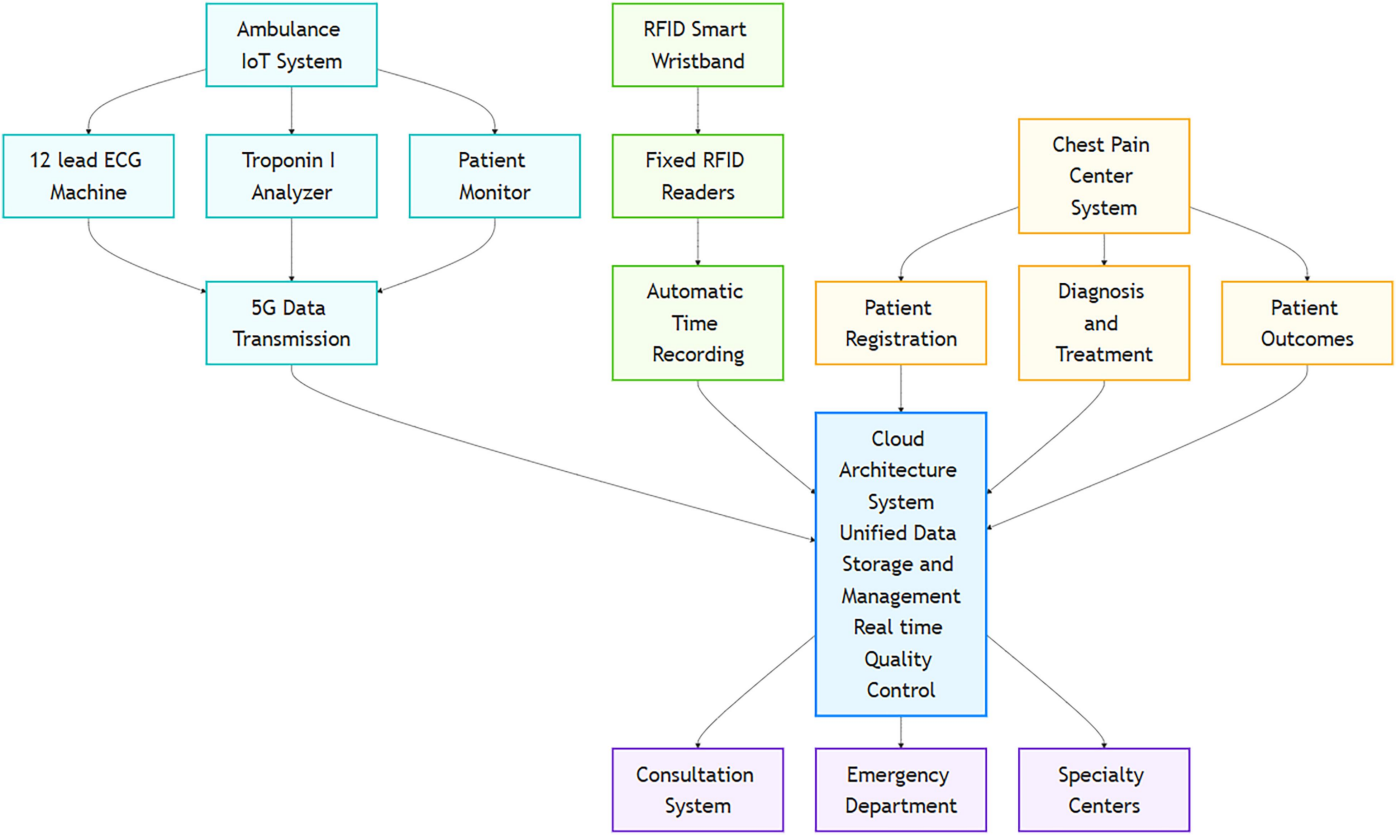
Schematic diagram of the “Smart + Specialty” integrated emergency platform.

1. Cloud Architecture System: Built on a cloud architecture system to uniformly store and manage data from the emergency process and transmit data to the in-hospital consultation system, emergency department, and specialty centers (chest pain specialty).
2. Ambulance Equipment: Ambulances are equipped with ECG machines, rapid detection devices for biomarkers, and an ambulance internet of vehicles system. The system integrated 12-lead ECG machines (Philips PageWriter TC70), portable troponin-I immunofluorescence analyzers (Getein 1100), and multi-parameter patient monitors to automatically collect core data such as ECG waveforms, troponin levels, electronic medical records, and vital signs (heart rate, blood pressure, oxygen saturation), transmitting it in real-time to the receiving hospital via a secure 5G data link.
3. RFID Smart Wristbands: Radio-frequency identification (RFID) smart wristbands (UHF band, compliant with ISO/IEC 18000-63 standard) can automatically collect the time of patient arrival at the emergency department entrance, resuscitation room, and other areas by being read by fixed RFID readers installed at these key nodes.
4. Specialty center system (chest pain center system): Includes functional templates for patient registration, pre-hospital emergency care, in-hospital treatment, chest pain diagnosis and treatment, and patient outcomes, achieving clinical informatization, real-time quality control management, and one-click data reporting through connection with in-hospital clinical information systems and medical devices. System architecture and data flow diagram was seen Figure 1.

#### 2.1.4 Integrated treatment process under the “smart + specialty” concept

1. After receiving a 120 emergency call, one pre-hospital emergency nurse is responsible for calming the patient's emotions over the phone and guiding self-rescue, while another accompanies the ambulance to assist in pre-hospital emergency care. The 120 emergency vehicle is equipped with troponin detectors, ECG monitors, audio-visual equipment, etc., creating a chest pain file for the patient after the first medical contact, quickly conducting tests such as troponin, ECG, aortic CTA, and echocardiography, and transmitting the results to the chest pain center system via fifth generation (5G) network. The vascular surgery attending physician quickly diagnoses based on the patient's ECG, CTA, vital signs, and test results, and feedbacks the diagnosis and treatment plan to the WeChat group. Emergency nurses prepare surgical instruments and drugs in advance. Pre-hospital emergency nurses explain the patient's condition, AoD treatment methods, and prognosis to the patient and family on the ambulance, alleviating their worries and helping them gain a preliminary understanding of the disease and surgical procedure. Patients are fitted with RFID smart wristbands to facilitate the recording of key timestamps. Upon arrival at the hospital, near field communication (NFC) technology and personal digital assistant (PDA) sensors automatically receive the patient and submit handover forms. Pre-hospital emergency nurses communicate with the family again, assist in signing the surgical informed consent form, and open a green channel in advance. Emergency nurses and vascular surgery specialty nurses assist doctors in carrying out surgical treatment. (2) For patients who come to the hospital on their own, emergency nurses complete the first ECG and aortic CTA within 10 min, send the results to the WeChat group, and immediately notify the vascular surgery attending physician to interpret the results and provide a treatment plan. If the patient is high-risk, immediate consent from the family is obtained, and standard procedures are initiated for surgical treatment.

#### 2.1.5 Optimization of the integrated treatment model

After surgery, team members analyze the entire emergency process and key timestamps recorded in the specialty center system, discuss issues in the emergency process, negotiate improvement measures, optimize emergency work, and ensure the quality of emergency nursing care.

### 2.2 Study design

This study employed a historical control design to evaluate the impact of a newly implemented “Smart + Specialty” integrated treatment model. The control group was retrospectively identified from consecutive patients who presented with aortic dissection and received the conventional emergency pathway between March 2022 and March 2023. Following the successful implementation of the new model, the study group was prospectively enrolled from consecutive eligible patients treated under the “Smart + Specialty” pathway between April 2023 and April 2024.

A total of 215 patients presented with chest pain emergencies and were suspected of having acute aortic dissection. Among them, 188 patients were diagnosed with acute aortic dissection based on clinical presentation and initial assessment. A total of 166 patients who met the inclusion criteria were finally enrolled and divided into two groups according to the order of their visits. The control group consisted of 81 patients who visited from March 2022 to March 2023, and the study group included 85 patients who visited from April 2023 to April 2024. This study was reviewed and approved by the hospital's ethics committee.

Inclusion criteria: meeting the diagnostic criteria for AoD (11); confirmed diagnosis of AoD by computed tomography angiography (CTA) and echocardiography; age >18 years; time of onset <6 h; normal cognitive function; signed informed consent.

Exclusion criteria: history of mental illness (*n* = 3); severe liver dysfunction (*n* = 2), kidney dysfunction (*n* = 2), or heart dysfunction (*n* = 4); chronic AoD (*n* = 5); death before hospital arrival or during transport (*n* = 4); co-occurring malignant tumors (*n* = 2); coagulation dysfunction (*n* = 2); communication barriers (*n* = 1); infectious diseases (*n* = 1).

### 2.3 Control and study groups

#### 2.3.1 Control group received standard emergency procedures

Upon receiving the emergency call, the ambulance team is immediately dispatched to provide pre-hospital emergency care. After the patient arrives at the hospital, the emergency nurse assesses the condition based on the patient's complaints, notifies experts for consultation, and quickly completes tests such as cardiac injury markers, electrocardiogram (ECG), aortic CTA, and echocardiography. Once the diagnosis and classification are confirmed, a green channel is opened, and corresponding surgical treatment is provided.

#### 2.3.2 Boundary conditions and scope of the intervention

The application and evaluation of the “Smart + Specialty” integrated model were governed by the following boundary conditions.

The intervention was exclusively applied to patients presenting during the defined study group period (April 2023–April 2024). Model activation was strictly for patients meeting the inclusion criteria (confirmed or highly suspected AoD with onset <6 h). Patients who died before hospital arrival or during transport were excluded from both the intervention and analysis. The model's functionality was contingent upon the operational status of its core components, including stable 5G connectivity, functional pre-hospital testing devices, and the cloud-based platform. Failure of these systems would result in reversion to the conventional protocol. The model was implemented and evaluated within the specific context of our single academic medical center, relying on its established resources, such as the multidisciplinary team and the pre-existing “green channel” for emergency surgery.

### 2.4 Observational indicators

1. Treatment Success Rate: Compare the treatment success rates between the two groups, the rate was calculated using the formula: Treatment Success Rate (%) = (Number of Successful Treatments)/(Total Number of Cases) × 100%. It refers to the situation where, after treatment, the patient's vital signs are stable, the symptoms related to aortic dissection (such as severe chest pain, etc.) are significantly relieved or disappear, and no death is caused by aortic dissection and its related complications during hospitalization.
2. Treatment Efficiency: Compare the triage time, diagnosis time, emergency stay time, referral time to intensive care unit (ICU), and time to symptom remission for successfully treated patients. Referral-to-ICU time was defined as the interval from the emergency physician's formal ICU transfer order to the patient's physical admission and monitoring setup in the ICU. Time to symptom remission was defined as the interval from the initiation of definitive treatment (surgical/endovascular) to the documented resolution of the primary presenting symptom (e.g., severe pain, NRS ≤ 1).
3. Adverse Event Rate: Compare the rates of secondary surgery, multiple organ failure, thrombosis, and infection for successfully treated patients.
4. Family Satisfaction with Nursing Care: The Newcastle Satisfaction with Nursing Scale (NSNS, Cronbach's α = 0.92) is used to survey family satisfaction with nursing care. The full mark ranges from 19 to 95 points, with 19–37 points, 38–56 points, 57–75 points, 76–94 points, and 95 points representing very dissatisfied, dissatisfied, neutral, satisfied, and very satisfied, respectively. Total satisfaction = very satisfied rate + satisfied rate.

### 2.5 Data completeness and analysis populations

This study employed two analytical datasets for evaluating different indicators. All enrolled patients (81 in the control group and 85 in the study group) were included in baseline characteristic analysis (following the intention-to-treat principle). For outcome assessments, treatment efficiency and safety analyses adopted per-protocol analysis, excluding 6 patients in the control group who did not complete the full treatment protocol (3 transfers, 2 surgery refusals, and 1 death during preoperative stabilization), with no exclusions in the study group. Nursing satisfaction analysis included all enrolled patients with a 100% questionnaire return rate. Baseline characteristic data were complete with no missing values, and all excluded cases resulted from definitive clinical events (non-random attrition), with no imputation performed for any missing data. To maintain analytical consistency, all time nodes related to treatment efficiency were automatically collected through the RFID/NFC system to ensure objectivity.

### 2.6 Statistical analysis

Data is analyzed using SPSS 26.0, with metric data represented as the mean ± standard deviation ($\bar{x}\pm s$) and tested with a *t*-test. Categorical data is represented as *n* (%) and tested with a chi-squared (*χ*^2^) test. A *P*-value less than.05 is considered statistically significant.

## 3 Results

### 3.1 Family satisfaction with nursing care

The overall family satisfaction with nursing care in the study group was 96.47% (82 out of 85), which was significantly higher than the control group's rate of 87.65% (71 out of 81), with a statistically significant difference (*P* *=* .04) see Table 1.

**Table 1:** Comparison of family satisfaction with nursing care between two groups [n (%）].

Item	Study group	Control group	*χ^2^*	*P*
Number of Cases	85	81		
Very Satisfied	35 (41.18)	28 (34.57)		
Satisfied	47 (55.29)	43 (53.09)		
Neutral	2 (2.35)	5 (6.17)		
Disatisfied	1 (1.18)	3 (3.70)		
Very Dissatisfied	0 (0.00)	2 (2.47)		
Total Satisfaction	82 (96.47)	71 (87.65)	4.466	0.04

Intention-to-treat analysis including all enrolled patients.

### 3.2 Patient enrollment and baseline characteristics

**Figure 2:**
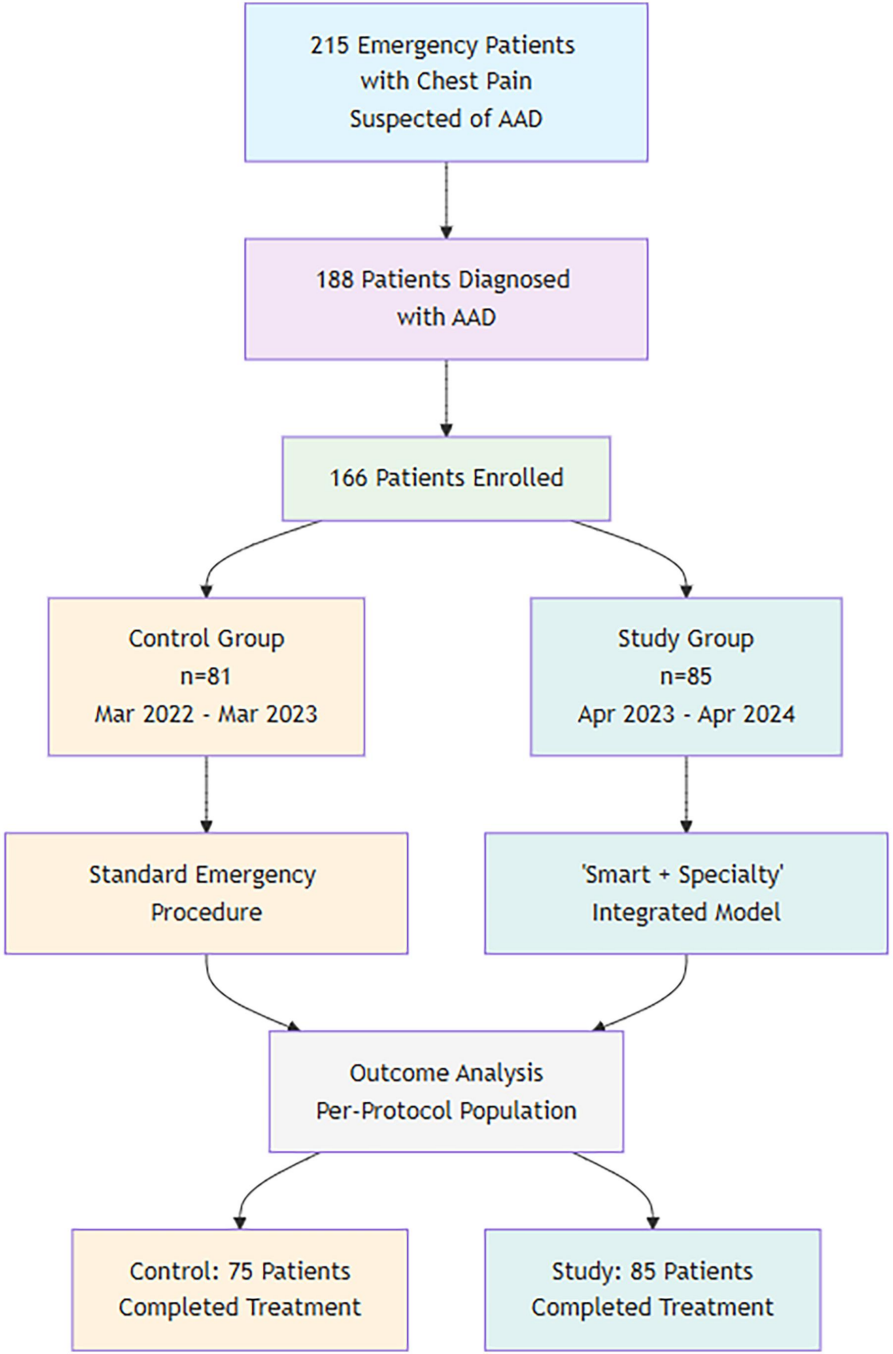
Study flowchart.

The patient enrollment process is detailed in Figure 2 (Study Flowchart). A total of 166 patients were included in the final analysis. The general data of the two groups were comparable (*P* > 0.05), as shown in Table 2.

**Table 2:** Comparison of general data between two groups.

Item	Study group (*n* = 85)	Control group (*n* = 81)	*t*/*χ*^2^/*u*	*P*
Demographics				
Gender (*n*, % Male)	65 (76.47)	60 (74.07)	0.128	.72
Age（$\bar{x}$±s, years)	65.95 ± 10.22	67.34 ± 11.12	0.839	.40
Underlying diseases (*n*, %)				
Hypertension	54 (63.53)	49 (60.49)	0.162	.69
Diabetes	19 (22.35)	14 (17.28)	0.669	.41
Hyperlipidemia	36 (42.35)	34 (41.98)	0.002	.96
Coronary artery disease	15 (17.65)	17 (20.99)	0.302	.58
Chronic kidney disease	8 (9.41)	10 (12.35)	0.380	.54
Stanford type (n, %)				
Type A	62 (72.94)	57 (70.37)	0.135	.71
Type B	23 (27.06)	24 (29.63)		
Anatomic & complication features (*n*, %)				
Aortic arch involvement	45 (52.94)	43 (53.09)	0.000	.99
Pericardial effusion	11 (12.94)	13 (16.05)	0.337	.56
Side branch involvement (n, %)				
Supra-aortic branches (Carotid/Subclavian)	18 (21.18)	16 (19.75)	0.052	.82
Visceral arteries (Celiac/SMA/Renal)	9 (10.59)	11 (13.58)	0.366	.55
Ischemic complications at presentation (n, %)				
Limb ischemia	7 (8.24)	9 (11.11)	0.422	.52
Mesenteric ischemia	3 (3.53)	4 (4.94)	0.087	.77
Myocardial ischemia	5 (5.88)	6 (7.41)	0.027	.87
Educational level (n)			0.113	.91
Junior high school and below	47 (55.29)	45 (55.56)		
High school/Technical secondary school	26 (30.59)	26 (32.10)		
College and above	12 (14.12)	10 (12.35)		
Admission vital signs ($\bar{x}$±s)				
Admission diastolic blood pressure (mmHg)	86.78 ± 3.85	87.12 ± 4.02	0.557	.58
Admission systolic blood pressure (mmHg)	150.27 ± 3.05	149.83 ± 2.87	0.956	.34
Heart rate (bpm)	92.4 ± 11.3	94.1 ± 12.7	0.956	.34

### 3.3 Treatment success rate

The study group achieved a 100% success rate (85/85), while the control group had a success rate of 92.59% (75/81). The comparison of treatment success rates between the two groups shows that the study group had a higher success rate than the control group, with a statistically significant difference (*χ*^2^ = 4.579, *P* = .03).

### 3.4 Treatment efficiency

The study group had shorter triage times, diagnosis times, emergency stay times, referral times to ICU, and time to symptom remission compared to the control group, with statistically significant differences (*P* *<* .001) see Table 3.

**Table 3:** Comparison of treatment efficiency between two groups ($\bar{x}$**±**s).

Item	Study group	Control group	*t*	*P*
Number of cases	85	75		
Triage time (min)	2.14 ± 0.51	6.02 ± 1.29	25.566	<.001
Diagnosis time (min)	24.57 ± 4.11	54.99 ± 6.86	34.475	<.001
Emergency stay time (min)	38.52 ± 7.63	86.38 ± 10.22	33.864	<.001
Referral to ICU time (min)	42.75 ± 6.55	87.36 ± 7.52	40.106	<.001
Time to symptom remission (h)	1.98 ± 0.51	3.02 ± 0.83	9.669	<.001

Per-protocol analysis. Data from six control group patients who did not complete the treatment protocol were excluded. All study group patients completed the protocol and were included.

### 3.5 Adverse event rate

The total adverse event rate in the study group was 3.53% (3 out of 85), which was significantly lower than the control group's rate of 12.00% (9 out of 75), with a statistically significant difference (*P* = .04). See Table 4. These events (e.g., secondary surgery for bleeding or stent graft issues, multi-organ failure, thrombosis) are multifactorial, being directly influenced by the patient's preoperative clinical status and intraoperative course.

**Table 4:** Comparison of adverse event rates between two groups [*n* (%)].

Item	Study Group	Control Group	*χ^2^*	*P*
Number of cases	85	75		
Secondary surgery	1 (1.18)	3 (4.00)		
Multi-organ failure	0 (0.00)	1 (1.33)		
Thrombosis	1 (1.18)	2 (2.67)		
Infection	1 (1.18)	3 (4.00)		
Total adverse events	3 (3.53)	9 (12.00)	4.121	0.04

The incidence of adverse events was calculated for patients who successfully completed the treatment protocol (per-protocol analysis). Thus, the six control group patients excluded from the success rate analysis were also excluded here, and the denominator for the control group is 75.

### 3.6 Surgical interventions and associated outcomes

The types of definitive surgical interventions performed are summarized in Table 5. In the study group, 58 patients (68.2%) underwent open surgical repair, while 27 patients (31.8%) were treated with TEVAR. In the control group, 55 of the 75 successfully treated patients (73.3%) underwent open repair, and 20 (26.7%) underwent TEVAR. The distribution of intervention types was not significantly different between the two groups (*P* = 0.50).

**Table 5:** Comparison of surgical interventions and associated adverse events [*n* (%)].

Item	Study group (*n* = 85)	Control group (*n* = 75)	*P*-value
Type of intervention			0.50
Open surgery	58 (68.2)	55 (73.3)	
TEVAR	27 (31.8)	20 (26.7)	
Adverse Events—open surgery	2/58 (3.4)	7/55 (12.7)	0.08
Adverse Events—TEVAR	1/27 (3.7)	2/20 (10.0)	0.57

The incidence of adverse events, stratified by the type of surgical intervention, is also detailed in Table 5. Within the study group, the adverse event rate was 3.4% (2/58) for open surgery and 3.7% (1/27) for TEVAR. In the control group, the adverse event rate was 12.7% (7/55) for open surgery and 10.0% (2/20) for TEVAR. This indicates that the “Smart + Specialty” model was associated with a lower incidence of adverse events compared to conventional care, and this benefit was consistent across both major types of surgical intervention.

## 4 Discussion

The integration of “smart” technologies characterized by automated data acquisition, real-time analysis, and instantaneous reporting is becoming a pivotal strategy in modern emergency medicine for time-critical conditions like aortic dissection. In this study, the term “smart” refers specifically to a system that operationalizes these principles through interconnected technologies (e.g., 5G-enabled ambulance devices, RFID tracking, and a cloud platform) to enable proactive clinical decision-making. Our “Smart + Specialty” model was developed within this conceptual framework to address systemic delays across the emergency care continuum.

Aortic Dissection (AoD) is a complex and critical cardiovascular disease (12–16). Studies have shown that if AoD does not receive timely and effective treatment, most patients can die within hours to weeks after onset (17). Therefore, optimizing the emergency process and improving the efficiency of AoD treatment is of great significance.

The conventional emergency process, with its separation between pre-hospital and in-hospital care, often lacks timely and effective communication, leading to treatment delays and critical patients missing the optimal rescue time (18, 19). Research indicates that building a smart emergency platform based on the internet can achieve pre-hospital to in-hospital medical linkage, enhancing treatment efficiency (20–22). In this study, the treatment success rate in the study group was higher than in the control group, and the time for each key node was shorter than in the control group, suggesting that the integrated treatment model under the “Smart + Specialty” concept can improve the treatment efficiency and success rate for AoD patients.

The key to this improvement lies in the fundamental differences between the conventional system and the “Smart + Specialty” model. The standard emergency system follows a conventional approach where patient care begins with ambulance dispatch providing basic life support, followed by sequential diagnostic tests and specialist consultations only after hospital arrival. In contrast, the “Smart + Specialty” model implements a proactive, technology-driven approach where a multidisciplinary team is immediately activated through digital platforms when the emergency call is received. Key differences emerge in several aspects: First, while the standard system relies on post-arrival diagnostics, the smart model enables real-time transmission of critical test results during patient transport through 5G-enabled devices. Second, where conventional care follows a linear workflow with time-consuming handoffs between departments, the integrated model allows parallel processing where diagnostics and treatment preparation occur simultaneously. Technologically, the standard system depends on paper-based documentation, whereas the smart approach utilizes automated tracking through RFID wristbands and NFC sensors, with all data integrated into a cloud-based platform. Furthermore, the conventional method offers limited pre-hospital communication, while the smart system initiates patient education and consent processes during ambulance transport. This comprehensive transformation from reactive to proactive care through integrated technology and team coordination results in significantly reduced time delays for critical interventions in aortic dissection cases.

The possible reason for this is that this study used a smart emergency system, achieving the digital transformation of ambulances and the integration of equipment information. Rapid examinations such as ECG on the ambulance can transmit results to the receiving hospital in a timely manner, realizing boarding as admission and information arriving at the hospital in advance. This helps in-hospital experts to diagnose the condition promptly, determine treatment plans, and prepare for treatment in advance, shortening the patient's waiting and diagnosis time in the hospital, and improving treatment efficiency and success rates. Additionally, the emergency team in this study was composed of collaborative diagnostic and treatment groups from disease-related departments and medical staff. Studies have shown that multidisciplinary collaborative nursing can provide patients with multidisciplinary and comprehensive diagnostic and treatment services, optimizing the quality of nursing services and further improving treatment outcomes (23). This study also defined the responsibilities of nursing staff, ensuring that nursing staff strictly adhere to the workflow, thereby improving their efficiency and reducing patient treatment time. At the same time, the smart emergency system can automatically record the full process of patient diagnosis and treatment data accurately, aiding the emergency team in timely analysis of treatment data, process optimization, and quality control of emergency care (24, 25).

The observed reduction in the total adverse event rate, while promising, requires careful interpretation. Adverse events such as those recorded are directly correlated with the patient's baseline clinical complexity and specific surgical interventions. Therefore, the lower rate in the study group cannot be attributed solely to faster response times. Instead, it is likely mediated by several indirect effects of the integrated model. By drastically shortening the door-to-intervention time and total ischemic time, the model may reduce the progression and severity of initial malperfusion syndromes, thereby mitigating the baseline risk for subsequent complications like multi-organ failure. Furthermore, the structured, multidisciplinary approach and homogenized training potentially contribute to more standardized perioperative care and earlier recognition of evolving problems, preventing their escalation into major adverse events. This suggests that the “Smart + Specialty” model's benefit on safety is not a direct function of speed alone, but a result of a more efficient, coordinated, and higher-quality system of care that influences the entire clinical pathway.

A particularly critical aspect of our findings concerns the application of the “Smart + Specialty” model to high-risk patients. As detailed in Table 1, the study and control groups were well-balanced at baseline, including the proportions of patients presenting with complex anatomic features (e.g., side branch involvement) and ischemic complications. These patients represent the most severe end of the AoD spectrum, for whom every minute of delay in definitive repair directly increases the risk of irreversible end-organ damage and mortality. The significantly shortened door-to-intervention times achieved by our model are, therefore, most consequential for this very cohort. The streamlined pathway—activating the specialist team, interpreting CTA, and preparing the operating room in parallel during pre-hospital transport—is designed precisely to mitigate the systemic delays that are most detrimental in these critical scenarios. Consequently, the observed reduction in adverse events and the 100% treatment success rate can be reasonably attributed, in significant part, to the model's efficacy in optimizing care for these highest-risk patients, powerfully underscoring its clinical value.

The results of this study also show that the adverse event rate in the study group was lower, indicating that the integrated treatment model under the “Smart + Specialty” concept in mitigating risks for AoD patients. While the established importance of early intervention in aortic dissection is well-recognized (26, 27), our model operationalizes this principle in the hyper-acute pre-hospital phase. By compressing the timeline from symptom onset to treatment, the protocol is designed to address the rapid clinical deterioration occurring in the initial golden hours. The association between the implemented model and reduced adverse events warrants further investigation to establish causality, but it aligns with the hypothesis that minimizing delays in this critical window is a decisive factor for patient outcomes. The integrated treatment model under the “Smart + Specialty” concept used in this study achieved the interconnection of pre-hospital and in-hospital emergency data through the internet, ensuring that patients can receive definitive diagnosis and treatment in the shortest possible time, reducing the risk of adverse events. The significantly reduced referral-to-ICU and symptom-remission times are direct clinical benefits of the streamlined pathway. The shorter ICU transfer time results from parallel processing and pre-notification via the digital platform, eliminating delays associated with sequential coordination. The rapid symptom relief is attributed to the drastically reduced total ischemic time, as expediting definitive treatment more quickly halts the ongoing dissection and restores perfusion, directly addressing the pathophysiological cause of the pain. Furthermore, this study formed an emergency team, conducted nursing training, and reviewed emergency work, effectively ensuring the quality of emergency nursing care and enhancing the nursing staff's capability for adverse event prevention, thereby reducing associated risks. In this study, the higher family satisfaction in the study group is likely attributable to its superior treatment efficiency and success rate.

## 5 Study limitations

This study has several limitations. First, the non-randomized, sequential allocation of patients introduces a significant risk of temporal confounding and selection bias, as unmeasured factors associated with the timing of implementation (e.g., seasonal variations, concurrent changes in staff expertise or hospital protocols) could influence the outcomes, despite attempts to control for these factors; Second, the single-center experience (*n* = 166) may limit generalizability to other settings; Third, the model's effectiveness depends on continuous 5G connectivity and specialized equipment availability; Fourth, performance improvements could partially reflect staff familiarity rather than technology alone; and the lack of long-term follow-up data restricts assessment of sustained outcomes—these constraints highlight the need for future multicenter randomized trials to validate the findings across diverse healthcare settings and evaluate cost-effectiveness. Fifth, as the management guidelines and clinical pathways for these two entities differ fundamentally—with Type A requiring emergent surgery and Type B often managed initially with medication—pooling their outcomes may confound the results. The observed benefits of the “Smart + Specialty” model, particularly in treatment success and efficiency, are likely driven predominantly by its impact on the time-sensitive Type A dissection cohort. This amalgamation limits the generalizability of our findings to each specific dissection type and may obscure the model's true effect on the medically managed Type B population.

## 6 Practical implications

The findings of this study, while preliminary, highlight several potential implications for clinical practice and future research. First, The dramatically reduced time to diagnosis and treatment directly translates to higher survival probabilities and lower risk of complications in a condition where minutes matter. The model appears to optimize the use of the critical early window after symptom onset. Second, this study describes a structured “smart emergency” pathway that integrates a cloud platform, mobile devices, and a team protocol. This framework could serve as a reference for other institutions seeking to develop similar systems for time-sensitive conditions like stroke or STEMI, though its effectiveness would require validation in those specific contexts. Third, the model demonstrates a potential shift from sequential processes to a more parallel workflow. It addresses the challenge of delayed specialist involvement and information gaps between pre-hospital and in-hospital teams, suggesting a direction for future system optimization to enhance teamwork and decision-making efficiency. Future research should focus on validating these findings in multicenter, randomized settings, evaluating long-term outcomes, and assessing the cost-effectiveness of this model.

## 7 Conclusion

In summary, this single-center study suggests that the integrated treatment model under the “Smart + Specialty” concept may improve treatment efficiency and success rate for emergency AoD patients, and could potentially reduce the risk of adverse events while increasing family satisfaction with nursing care. However, the generalizability of these findings is limited by the study's design and setting. The integrated treatment model under the “Smart + Specialty” concept has not yet been widely adopted in clinical practice, and further research is needed to confirm the effectiveness and safety of this treatment model.

## Data Availability

The original contributions presented in the study are included in the article/Supplementary Material, further inquiries can be directed to the corresponding author.

## References

[1] Zhu Y Lingala B Baiocchi M Tao JJ Toro Arana V Khoo JW Type A aortic dissection-experience over 5 decades: JACC historical breakthroughs in perspective. J Am Coll Cardiol. (2020) 76(14):1703–13. 10.1016/j.jacc.2020.07.061 33004136

[2] Khaja MS Williams DM. Aortic dissection: introduction. Tech Vasc Interv Radiol. (2021) 24(2):100745. 10.1016/j.tvir.2021.100745 34602274

[3] Hameed I Cifu AS Vallabhajosyula P. Management of thoracic aortic dissection. JAMA. (2023) 329(9):756–7. 10.1001/jama.2023.0265 36795378

[4] He J Zheng YL Zhao XY Xiao H Zhang JX Xiang DC. Implementation effects on regional coordination of aortic dissection based on emergency internet of things. Mil Med J S Chin. (2018) 32(4):230–3. 10.13730/j.issn.1009-2595.2018.04.004

[5] Mosconi MG Paciaroni M. Treatments in ischemic stroke: current and future. Eur Neurol. (2022) 85(5):349–66. 10.1159/000525822 35917794

[6] Liao Y Qi W Li S Shi X Wu X Chi F Analysis of onset-to-door time and its influencing factors in Chinese patients with acute ischemic stroke during the 2020 COVID-19 epidemic: a preliminary, prospective, multicenter study. BMC Health Serv Res. (2024) 24(1):615. 10.1186/s12913-024-11088-8 38730381 PMC11084012

[7] Fei XJ Pan HW Bao Y Ji CJ Wen XH. The impact analysis of the application of pre-hospital emergency information management software on the quality control time of severe trauma in-hospital emergency treatment. Chin J Emerg Med. (2024) 33(1):115–8. 10.3760/cma.j.issn.1671-0282.2024.01.021

[8] Kouroubali A Kondylakis H Logothetidis F Katehakis DG. Developing an AI-enabled integrated care platform for frailty. Healthcare (Basel). (2022) 10(3):443. 10.3390/healthcare10030443 35326921 PMC8948747

[9] Guo J Xu G Tian D Qu Z Qiu CW. A real-time self-adaptive thermal metasurface. Adv Mater. (2022) 34(24):e2201093. 10.1002/adma.202201093 35415933

[10] Li TT Kong LF Xiang T Luo D Peng CY Xie JB Research progress of pre-hospital emergency information integration construction based on 5G technology. Mod Instrum Med Treat. (2023) 29(1):2–6. 10.11876/mimt202301001

[11] Dong NG Zhu JM Liu JC Xiao YB Chen X Chen JM Chinese experts’ consensus of standardized diagnosis and treatment for aortic dissection. Chin J Thorac Cardiovasc Surg. (2017) 33(11):641–54. 10.3760/cma.j.issn.1001-4497.2017.11.001

[12] Sayed A Munir M Bahbah EI. Aortic dissection: a review of the pathophysiology, management and prospective advances. Curr Cardiol Rev. (2021) 17(4):e230421186875. 10.2174/1573403X16666201014142930 33059568 PMC8762162

[13] Reed MJ. Diagnosis and management of acute aortic dissection in the emergency department. Br J Hosp Med (Lond). (2024) 85(4):1–9. 10.12968/hmed.2023.0366 38708978

[14] Yang B. Does acute type A aortic dissection equal emergency aortic surgery? Ann Thorac Surg. (2023) 115(5):1093–4. 10.1016/j.athoracsur.2022.12.025 36572061

[15] Tanaka A Hebert AM Smith-Washington A Hoffstaetter T Goldenberg R Vemulapalli S Aortic dissection collaborative. Knowledge gaps in surgical management for aortic dissection. Semin Vasc Surg. (2022) 35(1):35–42. 10.1053/j.semvascsurg.2022.02.009 35501039

[16] Tekin G Tekin YK. Diagnosis of aortic dissection. Am J Emerg Med. (2024) 81:156. 10.1016/j.ajem.2023.02.004 36781374

[17] Zheng XW Su YX Zeng HX. Application of step-down thinking model combined with stage-targeted nursing in patients with aortic dissection. Qilu Nurs J. (2022) 28(3):128–32. 10.3969/j.issn.1006-7256.2022.03.040

[18] Wang X Ma S. Application effect of pre-hospital and in-hospital medical linkage system in emergency care of ischemic stroke patients. Chin J Clin Res. (2024) 37(3):485–8. 10.13429/j.cnki.cjcr.2024.03.035

[19] Jiang F Ye LP Sun KY Xie HX Zhao YY Wang R. Application of pre-hospital and in-hospital collaborative rescue platform in the venous thrombolysis for acute ischemic stroke. J Nurs Adv. (2022) 37(18):1682–4. 10.16821/j.cnki.hsjx.2022.18.011

[20] Hsieh JC Li AH Yang CC. Mobile, cloud, and big data computing: contributions, challenges, and new directions in telecardiology. Int J Environ Res Public Health. (2013) 10(11):6131–53. 10.3390/ijerph10116131 24232290 PMC3863891

[21] Tian Y Shen Z Zhao Y Zhou T Li Q Zhang M Design of an emergency medical information system for mass gatherings. Heliyon. (2024) 10(20):39061. 10.1016/j.heliyon.2024.e39061 PMC11620076 39640833

[22] Taylor RA Sangal RB Smith ME Haimovich AD Rodman A Iscoe MS Leveraging artificial intelligence to reduce diagnostic errors in emergency medicine: challenges, opportunities, and future directions. Acad Emerg Med. (2025) 32(3):327–39. 10.1111/acem.15066 39676165 PMC11921089

[23] Qiao YJ Bai XW Yuan FF Liu YM. Application of multidisciplinary collaborative nursing model in in-hospital transport of patients with aortic dissection. Qilu Nurs J. (2020) 26(8):35–7. 10.3969/j.issn.1006-7256.2020.08.009

[24] Zheng B Du X Wang J Xia B. Architecture design of wise medicine applied in mobile stroke emergency system based on 5G network technology. Chin J Stroke. (2021) 16(1):7–14. 10.3969/j.issn.1673-5765.2021.01.002

[25] Gao ZZ Yang J Zhu HL. Technical specifications for constructing an urgent and critical illness dataset based on health medical data. Chin J Crit Care Med. (2022) 42(6):461–9. 10.3969/j.issn.1002-1949.2022.06.001

[26] Huo J Xiao YL Yang X Wu ZY Zhang H. Analysis of the effect and safety of lumen reshaping after endovascular repair of Stanford B type aortic dissection at different intervention times. Natl Med J China. (2024) 104(17):1499–506. 10.3760/cma.j.cn112137-20240113-00098 38706057

[27] Wang Y Hong J Xu B Wang S He F. Successful treatment of a gravida with acute type A aortic dissection in the third trimester: a case report. Medicine (Baltimore). (2023) 102(13):e33423. 10.1097/MD.0000000000033423 37000058 PMC10063304

